# Direct anterior approach or posterior approach in total hip arthroplasty: A direct comparative study protocol

**DOI:** 10.1097/MD.0000000000022717

**Published:** 2020-10-16

**Authors:** Lingchao Kong, Long Chen, Li Sun, Xiaobin Tian

**Affiliations:** a Graduate School of Zunyi Medical University; b Department of Orthopaedics, Guizhou Provincial People's Hospital; c Department of Orthopaedics, Subsidiary Hospital of Guizhou Medical University, Guizhou, China.

**Keywords:** direct anterior approach, posterior approach, retrospective, study protocol, total hip arthroplasty

## Abstract

**Background:**

Two familiar surgical methods, posterior approach (PA) and direct anterior approach (DAA), have been extensively utilized in the treatment of total hip arthroplasty (THA) with similar long-term rates of success. The sufficient sample size and a good clinical trial are urgently needed. Considering the above factors, we implemented a retrospective research to compare the prognosis of patients with primary THA receiving the techniques of PA or DAA.

**Methods:**

This is an observational retrospective research that prospectively collected information via several surgeons at a center utilizing the 2 above treatment methods for unilateral primary total hip arthroplasty. A review of primary THA performed with DAA or PA between February 2017 and February 2019 was conducted in our hospital. The inclusion criteria contained the degenerative changes in end-stage of hip owing to the rheumatoid arthritis, inflammatory arthritis, and osteoarthritis, as well as the Crowe I and II dysplasia that did not require the enhancement. The primary endpoint was the Harris hip score. The measures of secondary outcome contained the operation time, length of incision, hospital stay, the complications after operation, as well as patient satisfaction. The Statistical Package for Social Sciences version 20.0 was utilizing for the statistical analysis (IBM Corporation, Armonk, NY).

**Results:**

We assumed that the 2 treatment methods possess similar results.

**Trial registration:**

This study protocol was registered in Research Registry (researchregistry6008).

## 1 Introduction

With the aging of the social population, the incidence rate of hip and knee osteoarthritis has increased rapidly, which has led to enormous economic and social burden. Total hip arthroplasty (THA), a gold standard of treatment for the end-stage treatment of osteoarthritis, including the removal of the affected hip joint and replacement with the artificial prosthesis (containing the acetabular and femoral parts).^[1–5]^

Two familiar surgical methods, posterior approach (PA) and direct anterior approach (DAA), have been extensively utilized in the treatment of THA with similar long-term rates of success. At present, posterior surgery is the most familiarly utilized THA surgical technique in the world, which adopts the gluteus maximus muscle to separate and then retain the gluteus minimus and gluteus medius.^[6–8]^ The DAA was modified on the basis of the Heuter method to enter hip joint through muscular space between the lumbar fascia muscles, sartorius, and the rectus femoris. In comparison with the PA, DAA is regarded to be a real muscle gap method, which can protect the soft tissue around hip joint, help keep the stability of hip joint, and then decrease the complications after operation.^[9–14]^

Conceptually, the anterior approach could lead to less damage than posterior approach because the anterior approach passes through the intermuscular and internerve plane without causing the muscle transection.^[15]^ Multiple reports have indicated that DAA was superior to the PA in the aspects of speed of recovery, hospital stay length, and blood loss after operation.^[16–18]^ Nevertheless, other researches have suggested that the DAA has a higher incidence of postoperative complications than is PA (for instance, femoral perforation, the femoral fractures during operation, and wound problems, particularly in the early stages of learning technique).^[19–21]^ Hence, in the literature, whether the therapeutic effect of DAA is better than that of PA is still controversial.

The sufficient sample size and a good clinical trial are urgently needed. Considering the above factors, we implemented a retrospective research to compare the prognosis of patients with primary THA receiving the techniques of PA or DAA. We assumed that the 2 treatment methods possess similar results.

## 2 Materials and methods

### 2.1 Study design and population

This is an observational retrospective research that prospectively collected information via several surgeons at a center utilizing the 2 above treatment methods for unilateral primary total hip arthroplasty. A review of primary THA performed with DAA or PA between February 2017 and February 2019 was conducted in our hospital. Institutional Review Board in the Subsidiary Hospital of Guizhou Medical University approved this study (ZY20201030). This current investigation has also been registered with the Research Registry (researchregistry6008).

The inclusion criteria contained the degenerative changes in end-stage of hip owing to the rheumatoid arthritis, inflammatory arthritis, and osteoarthritis, as well as the Crowe I and II dysplasia that did not require the enhancement. The exclusion criteria were the index of body mass index was ≥30 kg/m^2^; reluctance to take part in trial; with former hip surgery or use of hardware; infection; intolerance of general anesthesia; Crowe III and IV dysplasia of the hip.

### 2.2 Surgical procedures

All the DAA THAs were placed on the orthopedic traction table with supine position. The anterior incision starts from the posterior and distal 3 cm of the anterior superior iliac spine and then extended to about 10 cm above the muscle of tensor fascia lata. Then determine and develop the interval of Hueter to obtain the hip joint. The femoral neck resection was measured. The acetabulum was reamed and the components of acetabular were inserted. When preparing the femur, the legs should be straight, external rotation, and adduction. After the implantation of femur, the operating table was restored to the flat position. Ultimately, the remaining prosthesis was implanted for the wound closure and conventional capsular.

All the PA THAs were placed on the orthopedic traction table in lateral position. The incision was located in the center of the posterior surface of greater trochanter and the estimated length of each operation was recorded. On the basis of piriformis identification, the hip joint capsule and short external rotator muscle were labeled and then reflected. After the dislocation of hip joint, at templated level, femoral neck osteotomy was performed. Afterwards, the acetabulum was reamed and implanted with acetabular prosthesis. Ultimately, the remaining prosthesis was implanted for the wound closure and conventional capsular.

### 2.3 Postoperative care

On the basis of the agreement of health service, all the subjects were given thromboprophylaxis (low molecular weight heparin) and prophylactic antibiotics (intravenous gentamicin sulfate and cefazolin sodium). All the patients were given an identical standardized multimodal regimen pain, that is 4 doses of acetaminophen (1 g), 2 doses of 200 mg of celecoxib, and using morphine (first 48 hours) or tramadol (after 48 hours) to relieve pain.

The same rehabilitation scheme after operation was used in both groups. A standard program of rehabilitation, including walking aids and tolerable weight-bearing, was started the next day after surgery. When the surgical wound was stable, the hip joint abduction was 40°, the hip joint flexion was 100°, and the ability of daily activities was sufficient, the patients were discharged.

### 2.4 Outcome evaluation

The primary endpoint was the Harris hip score (HHS). HHS is reliable and effective and is general utilized as the gold standard or the reference for the evaluation of the structural validity of hip outcome measurements reported by other patients. HHS is a comprehensive measurement method, the score is between 0 and 100, which is seriously affected by function and pain; the higher the score, the better. It includes a total of 4 aspects: physical function (7 items; 47 points), pain (1 item; 44 points), and deformity (5 items; 5 points), as well as motion range (5 items; 4 points).

The measures of secondary outcome contained the operation time, length of incision, hospital stay, the complications after operation, as well as patient satisfaction. The level of satisfaction was assessed on the 100 mm horizontal visual analogue scale, in which 0 mm indicating complete satisfaction and 100 mm indicating complete dissatisfaction. The time of operation, hospital stay, and the length of incision were acquired from the database of our hospital, and paper records and electronic records. The postoperative complications and HHS were observed before and at least 2 years after the operation (Table 1).

**Table 1:**
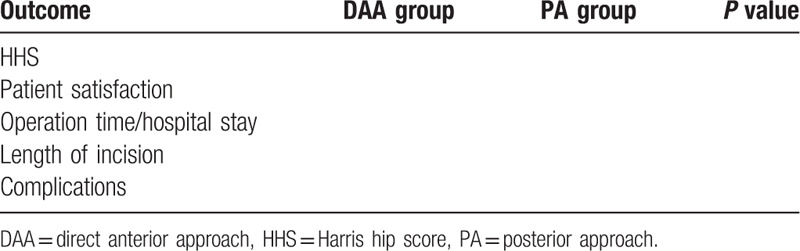
The postoperative outcomes in the 2 groups.

### 2.5 Statistical analysis and power analysis

The Statistical Package for Social Sciences version 20.0 was utilizing for the statistical analysis (IBM Corporation, Armonk, NY). The nonparametric tests and parametric tests are applied appropriately to evaluate the significant differences in continuous variables between the groups. The linear variables were compared between the groups with the Student *t* test. For the dichotomous variables, it can be evaluated with the Chi-square test. The regression analysis and multivariate linear analysis were applied for the determination of the independent prognostic predictor (postoperative HHS). When *P* is <.05, the difference is significant in statistics. The postoperative power calculation of HHS: in DAA group, there were 50 patients and in PA group, there were 210 patients. The minimum clinically significant difference was defined as 5 points, and the standard deviation was 9, and the power of alpha 0.05 was 0.81.

## 3 Discussion

In the past few years, more and more people have begun to pay attention to the minimally invasive, alternative methods in the total hip arthroplasty. Among the numerous possible methods, DAA is the only one that can really utilize the interval between muscles and nerves. The nerve-sparing and muscular-sparing nature of this method may allow a very rapid recovery after operation. Some large cases have revealed that the dislocation rate of anterior approach is low and walking can be restored early. Nevertheless, whether the anterior approach will be successful in improving postoperative recovery compared with other similar surgical options remains to be determined. The sufficient sample size and good clinical trial are urgently needed. Therefore, we implemented a retrospective research to compare the prognosis of patients with primary THA receiving the techniques of PA or DAA. We assumed that the 2 treatment methods possess similar results. The limitations of this current investigation contained the inherent limitations in any existing retrospective cohort research, involving the possibility of observation bias and selection. On top of that, we have a small sample size of 260 patients. Another limitation of our research is that the average follow-up period is only 2 years. And the in-depth follow-up is essential and is currently under way.

## Author contributions

**Conceptualization:** Lingchao Kong.

**Data curation:** Long Chen.

**Formal analysis:** Lingchao Kong.

**Funding acquisition:** Xiaobin Tian.

**Investigation:** Long Chen.

**Methodology:** Lingchao Kong.

**Project administration:** Xiaobin Tian.

**Resources:** Li Sun, Xiaobin Tian.

**Software:** Long Chen, Li Sun.

**Supervision:** Xiaobin Tian.

**Validation:** Li Sun.

**Visualization:** Li Sun.

**Writing – original draft:** Lingchao Kong.

**Writing – review & editing:** Xiaobin Tian.

## References

[1] Houdek MT Watts CD Wyles CC Total hip arthroplasty in patients with cerebral palsy: a cohort study matched to patients with osteoarthritis. J Bone Joint Surg Am 2017;99:488–93. 28291181 10.2106/JBJS.16.00528

[2] Dawson D Milligan D Callachand F Hip hemi-arthroplasty vs total hip replacement for displaced intra-capsular hip fractures: retrospective age and sex matched cohort study. Ulster Med J 2018;87:17–21. 29588551 PMC5849947

[3] Boyer B Bordini B Caputo D What are the influencing factors on hip and knee arthroplasty survival? Prospective cohort study on 63619 arthroplasties. Orthop Traumatol Surg Res 2019;105:1251–6. 31615748 10.1016/j.otsr.2019.07.020

[4] Jenkins DR Odland AN Sierra RJ Minimum five-year outcomes with porous tantalum acetabular cup and augment construct in complex revision total hip arthroplasty. J Bone Joint Surg Am 2017;99:e49. 28509833 10.2106/JBJS.16.00125

[5] Tian S Goswami K Manrique J Direct anterior approach total hip arthroplasty using a morphometrically optimized femoral stem, a conventional operating table, without fluoroscopy. J Arthroplasty 2019;34:327–32. 30448326 10.1016/j.arth.2018.10.023

[6] Petis S Howard JL Lanting BL Surgical approach in primary total hip arthroplasty: anatomy, technique and clinical outcomes. Can J Surg 2015;58:128–39. 25799249 10.1503/cjs.007214 PMC4373995

[7] Meermans G Konan S Das R The direct anterior approach in total hip arthroplasty: a systematic review of the literature. Bone Joint J 2017;99-B:732–40. 28566391 10.1302/0301-620X.99B6.38053

[8] Putananon C Tuchinda H Arirachakaran A Comparison of direct anterior, lateral, posterior and posterior-2 approaches in total hip arthroplasty: network meta-analysis. Eur J Orthop Surg Traumatol 2018;28:255–67. 28956180 10.1007/s00590-017-2046-1

[9] Barrett AA Ezzibdeh RM Horst PK Direct superior approach to the hip for total hip arthroplasty. JBJS Essent Surg Tech 2019;9:e17. 31579535 10.2106/JBJS.ST.18.00078 PMC6687490

[10] Wang Z Hou JZ Wu CH A systematic review and meta-analysis of direct anterior approach versus posterior approach in total hip arthroplasty. J Orthop Surg Res 2018;13:229. 30189881 10.1186/s13018-018-0929-4 PMC6127950

[11] Hart A Wyles CC Abdel MP Thirty-day major and minor complications following total hip arthroplasty-a comparison of the direct anterior, lateral, and posterior approaches. J Arthroplasty 2019;34:2681–5. 31358324 10.1016/j.arth.2019.06.046

[12] Malek IA Royce G Bhatti SU A comparison between the direct anterior and posterior approaches for total hip arthroplasty: the role of an ’Enhanced Recovery’ pathway. Bone Joint J 2016;98-B:754–60. 27235516 10.1302/0301-620X.98B6.36608

[13] Ozaki Y Baba T Homma Y Posterior versus direct anterior approach in total hip arthroplasty: difference in patient-reported outcomes measured with the Forgotten Joint Score-12. SICOT J 2018;4:54. 30480545 10.1051/sicotj/2018051 PMC6256966

[14] Cheng TE Wallis JA Taylor NF A Prospective randomized clinical trial in total hip arthroplasty-comparing early results between the direct anterior approach and the posterior approach. J Arthroplasty 2017;32:883–90. 27687805 10.1016/j.arth.2016.08.027

[15] Ponzio DY Poultsides LA Salvatore A In-hospital morbidity and postoperative revisions after direct anterior vs posterior total hip arthroplasty. J Arthroplasty 2018;33:1421–5. 29307677 10.1016/j.arth.2017.11.053

[16] Peters RM van Beers LWAH van Steenbergen LN Similar superior patient-reported outcome measures for anterior and posterolateral approaches after total hip arthroplasty: postoperative patient-reported outcome measure improvement after 3 months in 12,774 primary total hip arthroplasties using the anterior, anterolateral, straight lateral, or posterolateral approach. J Arthroplasty 2018;33:1786–93. 29502965 10.1016/j.arth.2018.01.055

[17] Jia F Guo B Xu F A comparison of clinical, radiographic and surgical outcomes of total hip arthroplasty between direct anterior and posterior approaches: a systematic review and meta-analysis. Hip Int 2019;29:584–96. 30595060 10.1177/1120700018820652

[18] Mjaaland KE Svenningsen S Fenstad AM Implant survival after minimally invasive anterior or anterolateral vs. conventional posterior or direct lateral approach: an analysis of 21,860 total hip arthroplasties from the norwegian arthroplasty register (2008 to 2013). J Bone Joint Surg Am 2017;99:840–7. 28509824 10.2106/JBJS.16.00494

[19] Panichkul P Bavonratanavech S Arirachakaran A Comparative outcomes between collared versus collarless and short versus long stem of direct anterior approach total hip arthroplasty: a systematic review and indirect meta-analysis. Eur J Orthop Surg Traumatol 2019;29:1693–704. 31363848 10.1007/s00590-019-02516-1

[20] Maldonado DR Laseter JR Kyin C Direct anterior approach in total hip arthroplasty leads to superior outcomes at 3-month follow-up when compared with the posterior approach: a matched study using propensity score analysis. J Am Acad Orthop Surg Glob Res Rev 2019;3:e19.00118. 10.5435/JAAOSGlobal-D-19-00118 PMC7004498 32072128

[21] Nelms NJ Birch CE Halsey DH Assessment of early gait recovery after anterior approach compared to posterior approach total hip arthroplasty: a smartphone accelerometer-based study. J Arthroplasty 2020;35:465–70. 31629624 10.1016/j.arth.2019.09.030

